# Prevalence of Leber hereditary optic neuropathy in the Community of Madrid (Spain), estimation with a capture-recapture method

**DOI:** 10.1186/s13023-024-03225-7

**Published:** 2024-05-29

**Authors:** María D. Esteban-Vasallo, M. Felicitas Domínguez-Berjón, Juan Pablo Chalco-Orrego, Julio González Martín–Moro

**Affiliations:** 1 Directorate General of Public Health, Regional Ministry of Health of Madrid, Madrid, Spain; 2 Department of Ophthalmology, University Hospital of Henares. Coslada, Madrid, Spain; 3 Faculty of Medicine, Universidad Francisco de Vitoria, Pozuelo de Alarcón, Madrid, Spain

**Keywords:** Leber Hereditary Optic Neuropathy, LHON, Hereditary optic neuropathy, Mitochondrial disease, G11778A, G3460A, T14484C, Positive predictive value, Prevalence, Rare diseases Registry

## Abstract

**Background:**

Leber hereditary optic neuropathy (LHON) typically presents in young adults as bilateral painless subacute visual loss. Prevalence data are scarce. The aim of this study was to examine the validity of different ascertainment sources used in population-based rare diseases registries to detect cases, and to explore the impact of a capture-recapture method in the estimation of the prevalence of LHON in the Autonomous Community of Madrid (ACM) in 2022.

**Methods:**

Descriptive cross-sectional population-based study. Potential LHON cases were detected by automatic capture from the healthcare information sources usually explored for the Regional Registry for Rare Diseases (SIERMA). Ophthalmologists provided data from their clinical registry. Positive predictive values (PPV) and sensitivity with 95% confidence intervals (CI) were estimated. Global and by sex prevalences were calculated with confimed cases and with those estimated by the capture-recapture method.

**Results:**

A total of 102 potential LHON cases were captured from healthcare information sources, 25 of them (24.5%) finally were confirmed after revision, with an overall PPV of 24.5% (95%CI 17.2–33.7). By source, the electronic clinical records of primary care had the highest PPV (51.2, 95%CI 36.7–65.4). The ophthalmologists clinical registry provided 22 cases, 12 of them not detected in the automatic capture sources. The clinical registry reached a sensitivity of 59.5% (95%CI 43.5–73.6) and the combination of automatic capture sources reached a 67.6% (95%CI: 51.5–80.4). The total confirmed cases were 37, with a mean age of 48.9 years, and a men: women ratio of 2.4:1. Genetic information was recovered in 27 cases, with the m.3460 mutation being the most frequent (12 cases). The global prevalence was 0.55 cases/100,000 inhabitants (95%CI 0.40–0.75), and with the capture-recapture method reached 0.79 cases/100,000 (95%CI 0.60–1.03), a 43.6% higher, 1.15 cases/100,000 (95%CI 0.83–1.58) in men and 0.43 cases/100,000 (95%CI 0.26–0.70) in women.

**Conclusions:**

The prevalence of LHON estimated in the ACM was lower than in other European countries. Population-based registries of rare diseases require the incorporation of confirmed cases provided by clinicians to asure the best completeness of data. The use of more specific coding for rare diseases in healthcare information systems would facilitate the detection of cases. Further epidemiologic studies are needed to assess potential factors that may influence the penetrance of LHON.

## Background

Leber hereditary optic neuropathy (LHON) is among the most common inherited mitochondrial diseases [1] and was the first to be associated with a primary mutation in mitochondrial DNA (mtDNA) [2]. Clasically three primary pathogenic point mitochondrial DNA mutations: G11778A, G3460A, and T14484C have been considered to be responsible for 90% of LHON cases [3]. Nevertheless in the last years the spectrum of the disease has been widened, as cases with no mitocondrial inheritance have been described, showing a greater genetic heterogeneity of nuclear LHON-like phenotypes [4–6].

LHON typically presents in young adults as bilateral painless subacute visual loss [3]. The diagnosis of this neuropathy should be based on a careful history and evaluation of key structural and functional visual parameters. Visual field and optical coherence tomography are key ancillary tests on the diagnosis of this neuropathy, but confirmation is made by the demonstration of a pathogenic DNA mutation [7].

Data on the prevalence of LHON in different populations are still sparse. According to Orphanet, the prevalence of the disease is estimated at 1.85/100,000–3.70/100,000 in Europe, but lower prevalence is reported in Serbia (0.19/100,000) [8]. There is no available data for Spain, although a recent study in the Autonomous Community of Madrid (ACM), based on surveys to neuro-ophthalmologists, estimated a prevalence between 0.65 and 0.94 cases in 100,000 inhabitants [9].

In 2015 the ACM implemented the Regional Registry for Rare Diseases (Sistema de Información de Enfermedades Raras de la Comunidad de Madrid, known by the acronym SIERMA) [10]. The purpose of this population-based disease registry is to study and analyze, from an epidemiological point of view, rare diseases in the region. It also participates in the National Registry of Rare Diseases (ReeR). SIERMA integrates data from different healthcare information systems: Minimum Basic Dataset at hospital discharge from public and private centres (MBDS), electronic clinical records in primary care (ECRPC), the mortality registry, the Madrid Registry of Renal Patients, data of consumption of orphan drugs (OD), data from the neonatal screening program for congenital metabolic and endocrine disorders, and voluntary notification from health professionals. In this last circumstance cases are confirmed directly while in the rest of them an automatic capture is carried out when certain criteria are met, and the clinical history is subsequently reviewed to validate the case. Classification and coding systems in current healthcare information systems frequently have not enough specificity to identify rare diseases [11], which may result in a lack of completeness, accuracy and comparability of cases.

High quality data of rare diseases registries is considered to be one of the most important element in the establishment and maintenance of a registry [12]. Capture-recapture models are commonly used for estimating prevalence in epidemiological studies, when information comes from incomplete lists [13, 14]. A recent review concluded that these models can be considered reliable to estimate the total number of cases with eye conditions using incomplete information from registers [15]. Estimation is based on the number of cases recorded on each registry, as well as the overlapping of cases between the registries. This allows to estimate the number of cases appearing in no list [13, 14].

The aim of this study was to examine the validity of different ascertainment sources used in population-based rare diseases registries, including the contribution of clinical registries, and to explore the impact of a capture-recapture method in the estimation of the prevalence of LHON in our region.

## Methods

Descriptive cross-sectional population-based study, perfomed in the ACM. The studied population was composed by those who have received healthcare in the ACM at some point during the period 2003–2022.

The procedure for detecting potential LHON cases in secondary data was implemented over all healthcare information sources usually explored for SIERMA. The period of data availability was different depending on the source of information. The MBDS is a mandatory database that contains all hospital admissions from public and private centres of the ACM since 2003. Up to 20 diagnosis are encoded at discharge using the International Classification of Diseases (ICD), the Clinical Modification of its ninth revisión (ICD-9-CM) until 2015, and the ICD-10-ES (stem from the ICD-10-CM-Clinical Modification) since 2016. All hospital discharges with a diagnosis coded as 377.16 between 2003 and 2015 or as H47.22 between 2016 and 2022 were obtained (both codes specific for hereditary optic atrophy).

In the ECRPC, healthcare episodes are coded as per the International Classification for Primary Care, 2nd edition (ICPC-2). Each code includes a descriptive text. LHON has no specific code in this classification and the eponym “Leber” is also present in the name of an uncommon form of retinitis pigmentosa (Leber congenital amaurosis). Therefore the stragegy adopted was the search in the descriptive text of episodes registered between 2009 and 2022 of the following texts: ‘LEBER’ and not ‘AMAUROSIS’, or ‘NOHL’, or ‘LHON’.

As idebenone (*Raxone®*) was approved in 2015 for the treatment of LHON by the European Medicines Agency under exceptional circumstances (was designated an orphan drug) [16], all patients in treatment with idebenone 150 mg (*Raxone®*) since 2016 (first year with data available) were considered also as potential cases.

No other potential case of LHON was detected from the rest of healthcare information systems routinely used for automatic capture for SIERMA. Case validation was performed by a detailed review of the complete electronic clinical records of the individual (primary care and hospital consultations, and hospital admissions) to achieve a classification of the potential case as: confirmed, discarded or mutation carrier. When no conclusive information was available or the confirmation was pending on, the case remained as possible. Information on mutations and family history was collected when available.

At the same time, the ophthalmologists investigating LHON in the ACM provided to SIERMA with data from their clinical registry of patients diagnosed with this condition until 2022.

Positive predictive values (PPV) and 95% confidence intervals (95%CI) were estimated for each automatic capture source for SIERMA and for their grouping as the fraction of LHON cases that fulfilled diagnostic criteria with respect to all potential LHON cases detected by the source/group of sources. Sensitivity and 95%CI were estimated as the fraction of confirmed LHON cases identified by each source and for the grouping of automatic capture sources for SIERMA, with respect to the total final number of confirmed cases detected in the study.

Sex and date of birth of each confirmed case was registered. Age at end of period was calculated. Mean and median age, and their respective 95%CI were calculated, total and by source of information. The prevalence of LHON in 2022 was calculated considering the patients detected with confirmed diagnosis and residing in Madrid for at least 6 months of that year. The total and by sex prevalences were recalculated from the cases estimated by the capture-recapture method, considering on the one hand the agrupation of automatic capture sources of potential cases for SIERMA and on the other the ophthalmologists clinical registry as two independent sources. The software used for the capture-recapture method was Epidat 3.1 It calculates the Petersen estimator, a formula that provides an estimate of the unknown number of individuals affected by a disease (within a population) when two list are available [15]. A lower overlap between lists translates into a greater difference between observed and estimated cases. We used the most recently published census data for the ACM to determine the point prevalence on 2022: 6,750,336 inhabitants living in the region, 48% of them being male.

The study was conducted by the Public Health regional authority; ethics approval and written patient consent were not required in accordance with current Spanish legislation on data protection.

## Results

A total of 102 potential cases of LHON were captured from healthcare information sources, 19 of them (18.6%) were identified in more than one source (Table 1). After revision, 25 (24.5%) of them were confirmed as diagnosed with LHON. Seven potential cases (6.9%) corresponded to asymptomatic mutation carriers, 2 were possible cases, and 68 (66.7%) were discarded as LHON cases (some of them diagnosed with other optic neuropathies).

**Table 1:** Potential LHON cases by source of information, true positives, positive predictive value (PPV) and sensitivity. Autonomous Community of Madrid, 2022

Source of ascertainment	Potential cases	Confirmed cases	Possible cases	Mutation carrier	Discarded cases	PPV (95%CI)	Sensitivity (95%CI)
Ophthalmologists clinical registry		22				100.0	59.5 (43.5–73.6)
Automatic capture sources for SIERMA	102	25	2	7	68	24.5 (17.2–33.7)	67.6 (51.5–80.4)
Case count by source*							
Electronic clinical records in primary care	43	22	2	4	15	51.2 (36.7–65.4)	59.5 (43.5–73.6)
Minimum basic data set at hospital discharge	37	7	1	2	27	18.9 (9.5–34.2)	18.9 (9.5–34.2)
Orphan drugs	41	6	0	1	34	14.6 (6.9–28.4)	16.2 (7.6–31.1)
Total		37	2	7	68		-

*Totals do not add up because a case can be captured by more than one source

PPV. positive predictive value, CI: confidence interval

**Fig. 1:**
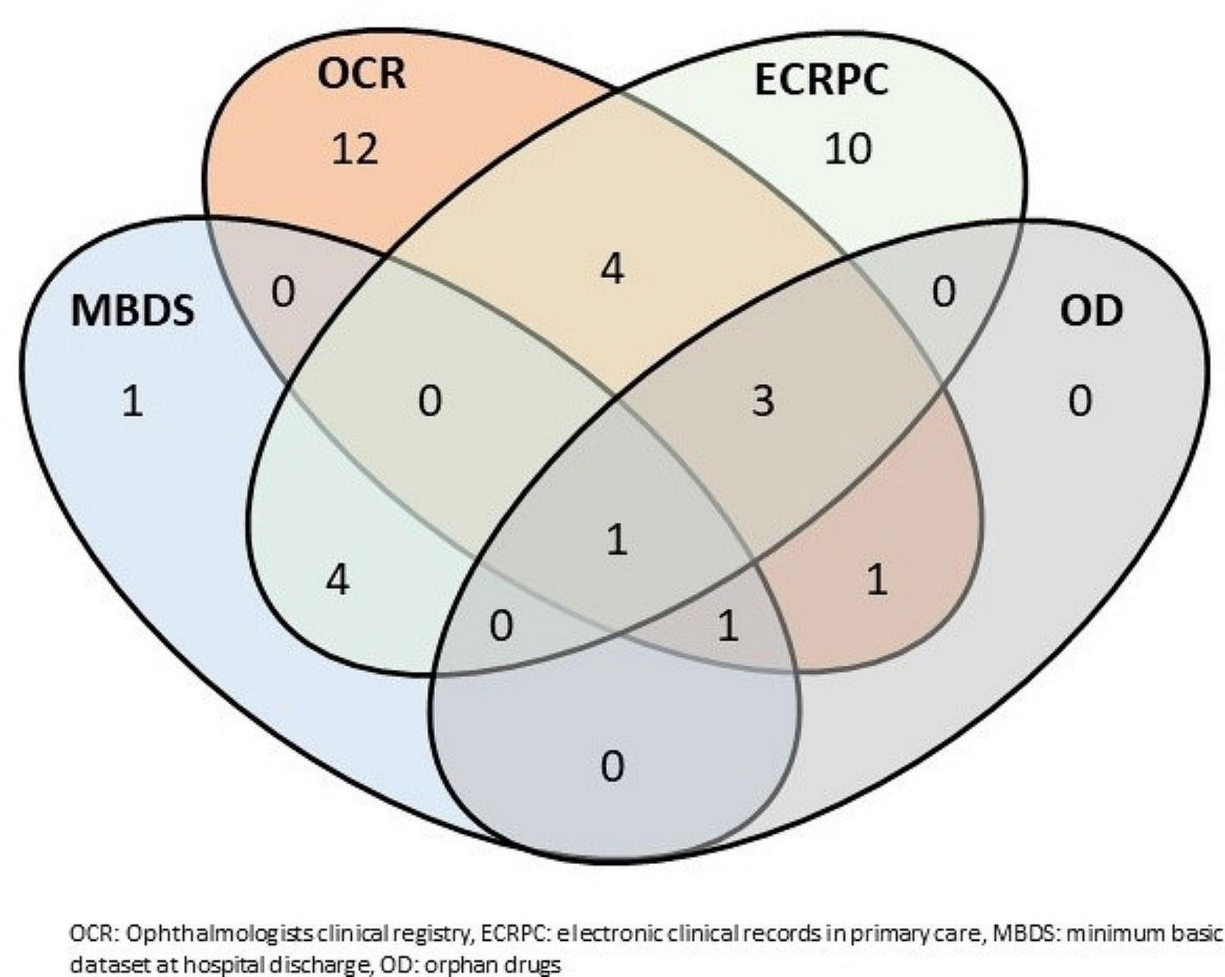
Venn diagram showing the overlaping LHON cases of the different sources of information. Autonomous Community of Madrid, 2022

Ophthalmologists provided data from their clinical registry on a total of 22 patients diagnosed with LHON until 2022. Less than a half of these cases (10, 45.4%) had been detected for SIERMA from the healthcare information systems used (Fig. 1), mainly from ECRPC, 8 cases, 3 of them also detected form the OD information system (*Raxone*®). In fact, the six confirmed cases detected from data of consumption of OD appeared also in the clinical registry of ophthalmologist. In total 12 cases provided by the ophthalmologists clinical registry had not been detected for SIERMA by the automatic capture healthcare information sources used, and 15 confirmed cases in SIERMA did not appear in the ophthalmologists clinical registry.

The automatic capture sources had a PPV of 24.5%, meaning most of the potential cases were not confirmed, and those potential cases identified through ECRPC were more likely to be confirmed. The similar sensitivity of ophthalmologist cases and automatically identified cases indicated that there was a similar proportion of true LHON cases missed by either method.

**Fig. 2:**
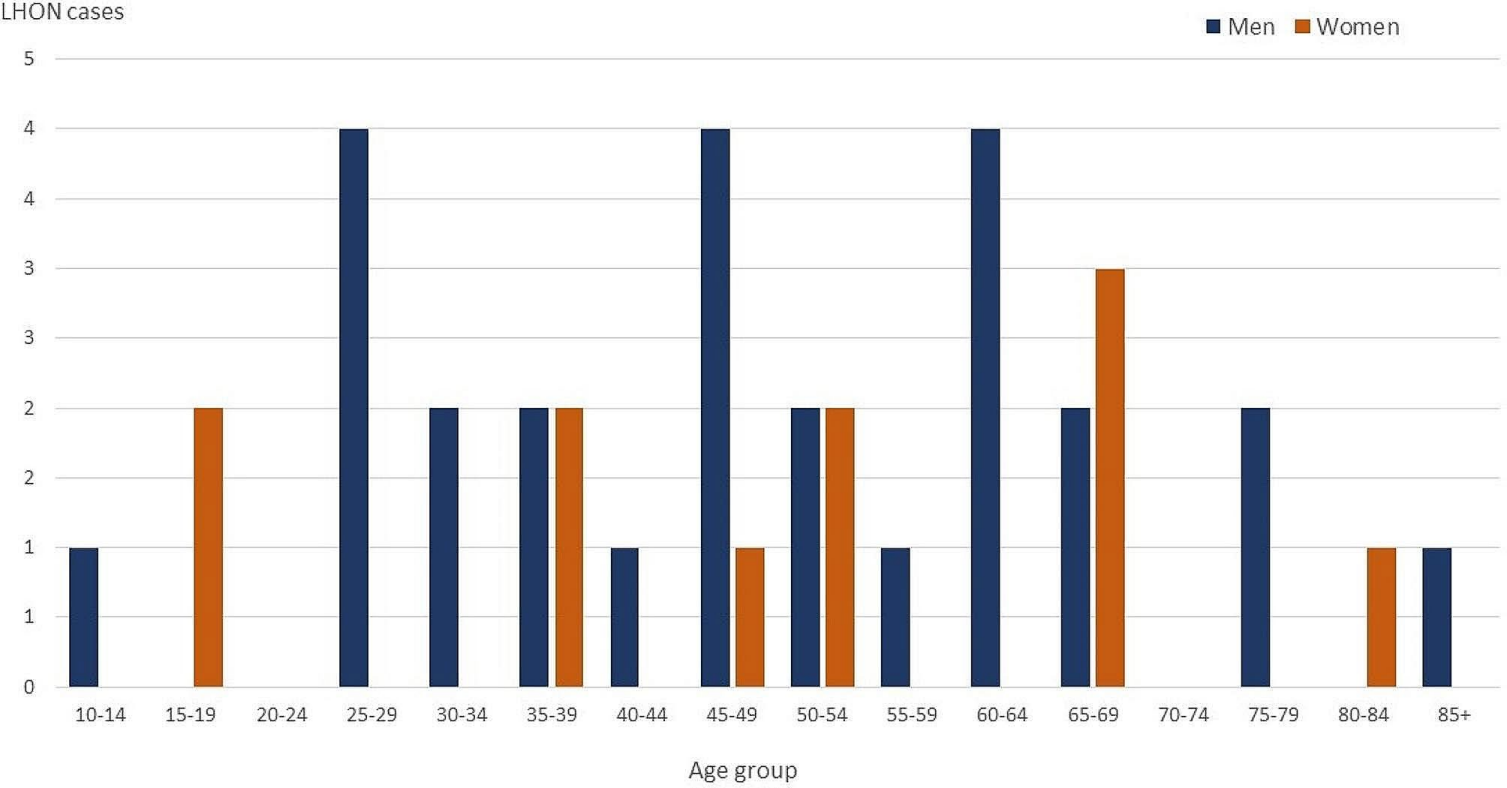
LHON cases by sex and age group at 31 December 2022. Autonomous Community of Madrid

**Fig. 3:**
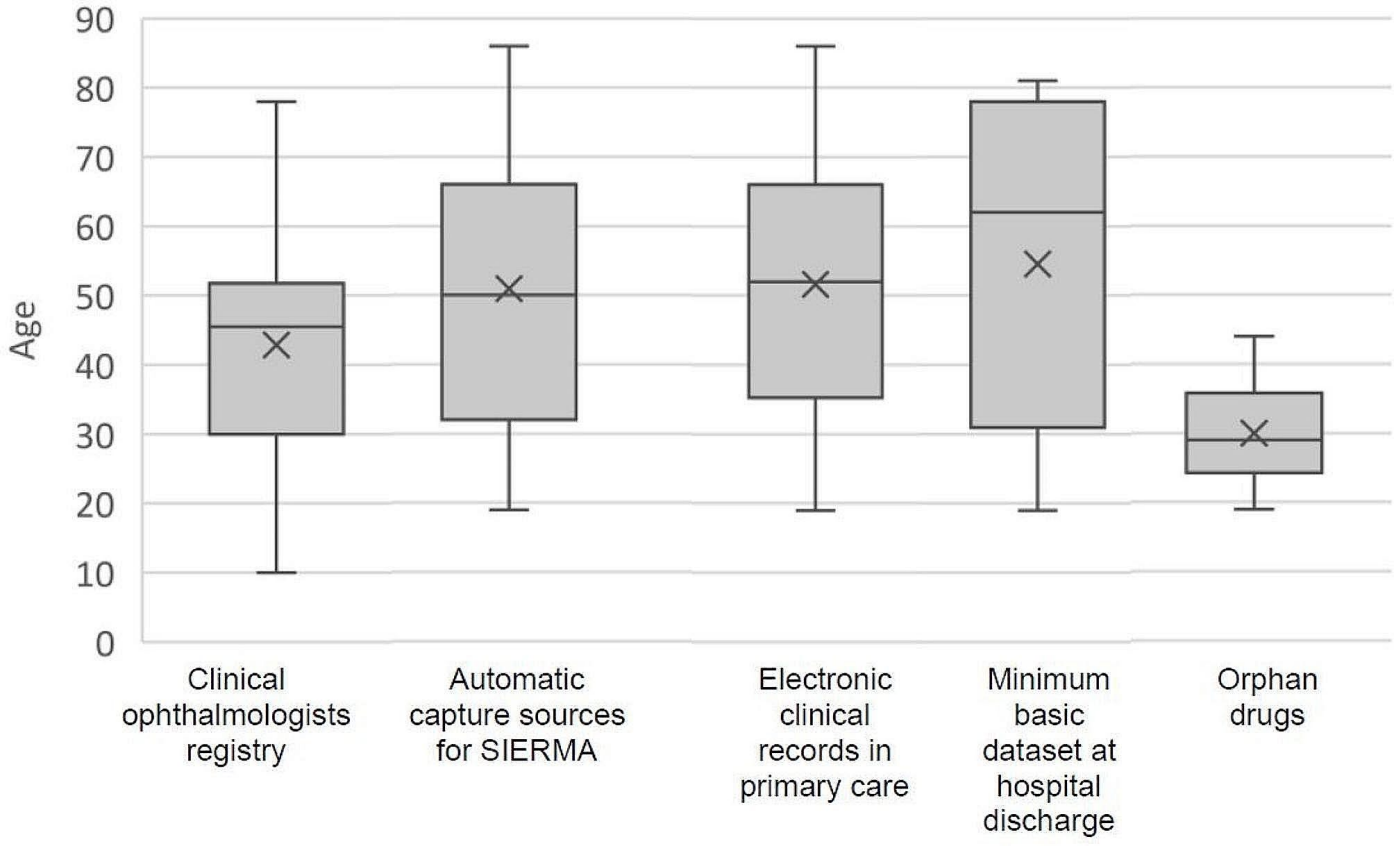
Age of LHON cases at 31 December 2022, by source of asceirtanment, Autonomous Community of Madrid

The age of LHON cases at the end of the period (31 December 2022) ranged from 10 to 86 years in men and from 18 to 81 years in women (Fig. 2), being the mean age 48.9 years (95%CI 42.5–55.2), with no differences by sex. The men: women ratio among the confirmed cases was 2.4:1. Differences in age and in sex distribution by source of detection are shown in Table 2; Fig. 3.

**Table 2:** Sociodemographic characteristics of prevalent LHON cases, by source, Autonomous Community of Madrid, 2022

	Cases	Ratio	Age at 31 December 2022	
		Men: women	Mean (95% CI)	Median (95% CI)
Ophthalmologists clinical registry	22	2.1:1	42.9 (35.1–50.7)	45 (33–51)
Automatic capture sources for SIERMA	25	2.6:1	51.0 (42.9–59.0)	50 (44–62)
Case count by source*:				
Electronic clinical records in primary care	22	2.7:1	51.6 (43.4–59.8)	52 (44–62)
Minimum basic data set at hospital discharge	7	1.3:1	54.6 (32.7–76.4)	62 (31–81)
Orphan drugs	6	5.0:1	30.0 (21.2–38.8)	29 (26–33)
Total	37	2.4:1	48.9 (42.5–55.2)	49 (44–60)

*Totals do not add up because a case can be captured by more than one source

CI: confidence interval

Patients detected from data of consumption of OD were significantly younger (median of 29 years) and among them the men:women ratio was the highest (5.0:1). None of the other data sources differed in age distribution. Genetic information was recovered in 27 cases (73.0%), 19 men and 8 women. The m.3460 mutation was the most frequent (12 cases), followed by the m.11778 mutation (7 cases). A family history of LHON was recorded in 17 cases (45.9%).

The global prevalence of LHON in the ACM in 2022 was 0.55 cases/100,000 inhabitants (95%CI 0.40–0.75) (Table 3). Prevalence in men reached 0.80 cases/100,000 (95%CI 0.55–1.18), being more than double than the prevalence in women (0.31 cases/100,000, 95%CI 0.17–0.56). The total cases estimated by the capture-recapture method were 53 (37 men and 15 women, ratio 2.5:1), leading to an estimated gobal prevalence of 0.79 cases/100,000 (95%CI 0.60–1.03), 1.15 cases/100,000 (95%CI 0.83–1.58) in men and 0.43 cases/100,000 (95%CI 0.26–0.70) in women.

**Table 3:** Cases and estimated prevalence of LHON, total and by sex. Autonomous Community of Madrid, 2022

	Cases	Rates per 100,000 (95%CI)	Estimated cases	Estimated rates per 100,000 (95%CI)
Men	26	0.80 (0.55–1.18)	37 (24–50)	1.15 (0.83–1.58)
Women	11	0.31 (0.17–0.56)	15 (8–22)	0.43 (0.26–0.70)
Total	37	0.55 (0.40–0.75)	53 (37–70)	0.79 (0.60–1.03)

CI: confidence interval

## Discussion

This study demonstrates the need to incorporate as many health information sources as possible to set a LHON population-based registry. When we compared the ability of different routinely collected healthcare datasets to identify all cases with LHON, we found that a considerable number of patients were detected only in one of them. A comprehensive method to find cases from multiple imperfect real-world data sources is important to demonstrate that the conclusions are complete and valid for prevalence estimates.

Potential cases identified from ECRPC had the greatest likelihood of being confirmed cases, highlighting how primary care doctors take care allocating labels like LHON and specifications like mutation carrier status. Optic atrophy ICD codes from hospital discharges were not specific for LHON, coding several autosomal dominant or recessive rare diseases. LHON does not require hospitalization by itself, so if the patient has no hospital admissions or the LHON is not registered as a complementary diagnosis at hospital discharge, the case goes unnoticed for the implemented detection system. Patients detected by searches in ECRPC and MBDS have a higher age, as older patients are more likely to visit the outpatient clinic and to be admitted to hospitals.

Some studies have shown that idebenone may help to recover lost vision or to maintain good residual vision in LHON [17, 18]. The search oriented by the prescription of this drug had a very low sensitivity since this drug was recently approved and has a narrow time window.

When we considered the ophthalmologists clinical registry, the lack of completeness in both population and clinical registries becomes evident. The clinical registry incorporates cases as they are diagnosed and in successive clinical reviews. It is likely that in this disease, once severe vision loss is established and therapeutic options have run out, the patient can be lost to follow-up. The clinical registry probably offers a better estimate of the incidence than of the prevalence of the disease. The fact that all the cases identified from data of consumption of idebenone, although only six, are markedly younger and predominantly male, and all of them appear in the ophthalmologists clinical registry, seems to indicate that these are recently diagnosed cases. On the contrary, the SIERMA cases obtained from ECRPC and MBDS have higher ages, capturing confirmed cases not registered in the ophthalmologist clinical registry.

Combining the clinical registry (mostly new cases) and automated capture from primary care (older longstanding cases) provides a more complete picture as demonstrated by the capture-recapture method. This method estimated a prevalence 43.6% higher than the calculated with available data. However, the estimated prevalence, 0.79/100,000 (0.60–1.03) was nearly three times lower than the 2.3/100,000 estimated by Orphanet in Europe [8]. A recent study in the ACM with a different methodology obtained similar results, estimating a prevalence of LHON also lower, between 0.65–0.94/100,000 inhabitants [9]. Different studies in European regions have published higher prevalences, the minimum point prevalence of visual failure due to LHON in North East of England was 3.22 per 100,000 [19], although based on the adult population younger than 65 years and consequently not directly comparable with other prevalence data. The prevalence in Denmark and Finland is very similar, 1.85/100,000 and 2.00/100,000, respectively [20, 21] and even higher in the Dutch population (2.56/100,000) [22]. However, a study performed in Serbia estimated a much lower prevalence, with a minimum point prevalence of 0.19/100,000 [23]. A meta-analysis of the LHON prevalence in Europe estimated a frequency of 2.22/100,000, but suggested that further epidemiologic studies, particularly in countries of southern and Eastern Europe, were needed to provide a more accurate prevalence estimate and also to identify potential hot spots of LHON disease in Europe [24]. Wordlwide, the estimated prevalence of LHON seems to be of the same magnitude than in northern European countries, 1.97/100,000 in Japan [25], 1.46/100,000 in Australia [26].

The lower prevalence detected in our study could be due to various reasons. In other centers with known pedigrees of LHON, the genetic testing and case identification are enhanced, and publication bias highlights these centers over other cities. Our method may have also missed some cases of LHON, not fully addressed by the capture-recapture calculation method. It is also possible that mitochondrial haplogroups or differences in other factors affecting penetrance or mutation carrier rates are indeed different in the Spanish population compared to other parts of Europe. The frequency of the different primary LHON mutations varies throughout the world, and their prevalence is unknown in our country, but LHON mutations have been detected at higher than expected prevalence in large genetic cohorts, suggesting a very low penetrance [27]. The fact that the reported incidence is higher in northern countries make us suggest that haplotype distribution or unidentified latitude related environmental factors may influence the penetrance of this disease.

The male to female ratio was 2.4:1, lower than that described in other European populations, in which the male predominance varies significantly among affected patients: 3.3:1 in North East England, 3.4:1.0 in Finland, 3.7:1 in Denmark, 5.4:1 in the Netherlands and 6:1 in Serbia [19–23]. Out of Europe even higher ratios have been described, like 8.2:1 in Japan and 8.2:1 India [25, 28]. The ratios vary also for each of the primary LHON mutations [26], and the higher proportion of m.3460 mutation among our cases could partially explain the reduced male predominance [29]. Although the different study methodologies used may limit the comparability of these data, a large international study had already raised doubts about the traditional 5:1 male-to-female ratio commonly reported in the literature, as they found a 3:1 ratio, more similar to our data [30]. The lower than expected male to female ratio reinforces the validity of our results. If ophthalmologist were able to diagnose atypical cases in females, they should have been able to diagnose the typical cases in males. The factors speculated for this gender differences are the nuclear modifier genes on the X-chromosome [31] and also circulating estrogens in women could play a role [32].

### Limitations

The sources of information are mainly from the public health system, so there could be lack of cases assisted only in private healthcare centers, but under the ophthalmologists perception most patients with rare diseases, especially the severe and disabling ones, are diagnosed, followed and treated in the public health system [9]. Data from genetic laboratories were not available for automatic capture. The period for capturing potential cases could be insufficient for an accurate detection of all prevalent cases.

## Conclusions

The estimated prevalence of LHON in the ACM was lower than the reported in other European countries. Nevertheless, the concordance of this results with the ones obtained in a recent study using incidence in our region to calculate prevalence and the low female to male ratio reinforce the validity of these results. Population-based registries of rare diseases require the incorporation of confirmed cases provided by clinicians to asure the best completeness of data. The implementation of more specific coding for rare diseases, as ORPHAcodes, would also facilitate the capture, validation, monitoring and comparability of cases. The capture-recapture method allows to estimate the missing cases and to make more reliable estimations of prevalence. Maintaining surveillance systems over time seems necessary to generate more valid data. Further epidemiologic studies in countries of southern Europe are needed to provide a more accurate prevalence estimate of LHON in different populations and to assess possible factors that may influence the penetrance of the disease.

## Data Availability

The data that support the findings of this study are not available due to confidentiality reasons, acording to current local legislation [10].

## Abbreviations

ACM: Autonomous Community of Madrid
ECRPC: Electronic clinical records in primary care
ICD: International Classification of Diseases
ICD-9-CM: International Classification of Diseases, ninth revisión, Clinical Modification
ICD-10-ES: International Classification of Diseasesm tenth revision, Clinical Modification, Spanish version
ICPC-2: International Classification for Primary Care, 2nd edition
LHON: Leber hereditary optic neuropathy
MBDS: Minimum Basic Dataset at hospital discharge
OCR: Ophthalmologhysts clinical registry
OD: Orphan drugs
SIERMA: Sistema de información de enfermedades raras de la Comunidad de Madrid (Regional Registry for Rare Diseases)
